# Supplementary material for: A type 1 immunity-restricted promoter of the IL−33 receptor gene directs antiviral T-cell responses
Source: Nat Immunol. 2024 Jan 3;25(2):256–67. doi: 10.1038/s41590-023-01697-6 (PMC10834369; doi:10.1038/s41590-023-01697-6)
Supplement: Supplementary file 1 — Reporting Summary [file 41590_2023_1697_MOESM1_ESM.pdf]

nature portfolio

Corresponding author(s): Max Löhning, Tobias Brunner

Last updated by author(s): Oct 26, 2023

Reporting Summary

Nature Portfolio wishes to improve the reproducibility of the work that we publish. This form provides structure for consistency and transparency in reporting. For further information on Nature Portfolio policies, see our Editorial Policies and the Editorial Policy Checklist.

Statistics

For all statistical analyses, confirm that the following items are present in the figure legend, table legend, main text, or Methods section.

n/a	Confirmed	
☐	☒	The exact sample size (*n*) for each experimental group/condition, given as a discrete number and unit of measurement
☐	☒	A statement on whether measurements were taken from distinct samples or whether the same sample was measured repeatedly
☐	☒	The statistical test(s) used AND whether they are one- or two-sided<br>*Only common tests should be described solely by name; describe more complex techniques in the Methods section.*
☒	☐	A description of all covariates tested
☐	☒	A description of any assumptions or corrections, such as tests of normality and adjustment for multiple comparisons
☐	☒	A full description of the statistical parameters including central tendency (e.g. means) or other basic estimates (e.g. regression coefficient) AND variation (e.g. standard deviation) or associated estimates of uncertainty (e.g. confidence intervals)
☐	☒	For null hypothesis testing, the test statistic (e.g. *F*, *t*, *r*) with confidence intervals, effect sizes, degrees of freedom and *P* value noted<br>*Give P values as exact values whenever suitable.*
☒	☐	For Bayesian analysis, information on the choice of priors and Markov chain Monte Carlo settings
☒	☐	For hierarchical and complex designs, identification of the appropriate level for tests and full reporting of outcomes
☐	☒	Estimates of effect sizes (e.g. Cohen's *d*, Pearson's *r*), indicating how they were calculated

Our web collection on statistics for biologists contains articles on many of the points above.

Software and code

Policy information about availability of computer code

Data collection

FACS Aria, Aria II and LSRFortessa flowcytometers (BD); MACSQuant (Miltenyi Biotec); Immunospot (CTL); QuantStudio 7 (Thermo Scientific); Diva software v8.1 (BD); Chromium Controller (10x Genomics); Qubit 2.0 Fluorometer (ThermoFisherScientific) ; Fragment Analyzer (Agilent); NextSeq500 (Illumina); NextSeq2000 (Illumina)

Data analysis

Flow cytometry data were analyzed using FlowJo (v10.7.1). Statistical analysis was performed with Graphpad Prism (v.10.0.3).
Analysis of sequencing data was performed using the following packages:

cellranger_3.1.0,
R_4.2.2,
Seurat_4.0.0,
immunarch_0.6.6,
TopHat_2.1.1,
Bowtie_2.2.8.0,
featureCounts_1.5.1,
DESeq2_1.36.0,
AnnotationDbi_1.58.0
EnhancedVolcano_1.14.0,
ComplexHeatmap_2.12.1,
pheatmap_1.0.12,
ggplot2_3.4.0,
clusterProfiler_4.4.4,
hisat2_2.2.1,
cufflinks_2.2.1,
proActiv_1.6.0,

CrossMap_0.5.2
Integrated Genome Viewer (Snapshot 12/21/2019 and 2.16).

Code related to the data analysis and R session information of this study have been deposited to GitLab. (https://agloehninggitlab.gitlab.io/BrunnerServe-Type1-ST2/).

For manuscripts utilizing custom algorithms or software that are central to the research but not yet described in published literature, software must be made available to editors and reviewers. We strongly encourage code deposition in a community repository (e.g. GitHub). See the Nature Portfolio guidelines for submitting code & software for further information.

Data

Policy information about availability of data

All manuscripts must include a data availability statement. This statement should provide the following information, where applicable:

- Accession codes, unique identifiers, or web links for publicly available datasets
- A description of any restrictions on data availability
- For clinical datasets or third party data, please ensure that the statement adheres to our policy

Generated single cell and bulk RNA-Seq data are provided via the Gene Expression Omnibus (GEO) under accession codes GSE204695 and GSE204693. Published data are accessible via the following GEO accession codes: Chip-Seq: GSM550303 (STAT4), GSM998272 (T-bet), GSM523226; (GATA-3) GSM776557 (human T-bet); ATAC-Seq: GSE120532 (CD4+ T cells), GSE111902 (CD8+ T cells). PhyloP conservation tracks are provided by the UCSC Sequence and Annotation database https://hgdownload.soe.ucsc.edu/goldenPath/mm10/phyloP60way/. Mast cell and ILC2 RNA-Seq data are available at the NCBI Sequence read archive via Run ID SRR7549295 (ILC2s) and SRR6155875 (mast cells). FANTOM5 CAGE-Seq data and CAGE-associated transcript data are available from the FANTOM5 collection (https://fantom.gsc.riken.jp/5/). Genome releases GRCm38.p6 and GRCh37.p13 are accessible at Ensemble (http://www.ensembl.org/index.html). Mouse data can be inspected in the UCSC genome browser: https://genome.ucsc.edu/s/agloehning/BrunnerServeetal2023. Source data are provided with this paper.

Field-specific reporting

Please select the one below that is the best fit for your research. If you are not sure, read the appropriate sections before making your selection.

☒ Life sciences ☐ Behavioural & social sciences ☐ Ecological, evolutionary & environmental sciences

For a reference copy of the document with all sections, see nature.com/documents/nr-reporting-summary-flat.pdf

Life sciences study design

All studies must disclose on these points even when the disclosure is negative.

Sample size	No specific statistical tests were performed to predetermine the sample size. Sample size was chosen based on previous experiments (Bonilla et al. Science 2012; Baumann et al. PNAS 2015; Baumann et al. Frontiers in Immunology 2019) and availability of mice with the appropriate genotype. Sample size for each experiment is indicated in the legend.
Data exclusions	Low quality cells and biologically irrelevant cells types were removed during the sc-RNASeq analysis as follows: Features with correlation coefficients >0.85 to Gm42418, Malat1, AY036118 and Lars2 were removed from the count matrix. Hashtag demultiplexing (representing the 3 biological replicates per genotype) was performed based on Seurats HTODemux with the parameter positive-quantile at 0.99. Doublets and untagged cells were filtered out. Cells with expression values for Cd8a or Cd8b1 and Cd3g, Cd3d or Cd3e, with >200 and <4500 features and <10% UMI for mitochondrial genes were kept for further analysis.After ranking by residual variance, 3000 variable genes were determined. The genes encoding TCR variable regions (Trav, Trbv, Trdv, Trgv) were removed for further analyses.
Replication	Experiments were performed with multiple biologically independent samples. The number of independent samples (n) and the number of independent repetitions for each experiment are indicated in the legend.
Randomization	Allocation of mouse samples was not randomized as mice were age- and sex-matched and allocated to experimental groups based on genotyping results. Human buffy coats of heathly patients were obtained from the German Red Cross (DKR Berlin) without information about gender or age. Human CTLs, Th1, and Th2 cells were randomly sorted from individual donors.
Blinding	For experiments involving murine and human samples, investigators were not blinded as the investigators who performed the experiment also planned them. Additionally, for murine experiments, genotyping was required for group allocation.

Reporting for specific materials, systems and methods

We require information from authors about some types of materials, experimental systems and methods used in many studies. Here, indicate whether each material, system or method listed is relevant to your study. If you are not sure if a list item applies to your research, read the appropriate section before selecting a response.

Materials & experimental systems

n/a	Involved in the study
☐	☒ Antibodies
☐	☒ Eukaryotic cell lines
☒	☐ Palaeontology and archaeology
☐	☒ Animals and other organisms
☐	☒ Human research participants
☒	☐ Clinical data
☒	☐ Dual use research of concern

Methods

n/a	Involved in the study
☒	☐ ChIP-seq
☐	☒ Flow cytometry
☒	☐ MRI-based neuroimaging

Antibodies

Antibodies used

The following antibodies have been used for flow cytometry analysis and sorting (Antibody, Conjugate, Clone, Provider and Cat#, Dilution):
Rat monoclonal anti-mouse CD25, Biotin, 7D4, eBioscience Cat#13-0252-85, 1:200
Hamster monoclonal anti-mouse CXCR3, Biotin, CXCR3-173, eBioscience Cat#13-1831-82, 1:500
Hamster monoclonal anti-mouse CXCR3, PerCP-Cyanine5.5, CXCR3-173, eBioscience Cat#45-1831-82, 1:100
Mouse monoclonal anti-mouse/human CD3, PerCP-eFluor710, 17A2, eBioscience Cat#46-0032-82, 1:300
Rat monoclonal anti-mouse CD90.2 (Thy1.2), PE, 30-H12, Biolegend Cat#105308, 3µg i.v.
Rat monoclonal anti-mouse CD4, Biotin, RM4-5, eBioscience Cat#13-0042-82, 1:300
Rat monoclonal anti-mouse CD4, PerCP-Cyanine5.5, RM4-5, eBioscience Cat#45-0042-82, 1:400
Rat monoclonal anti-mouse CD4, APC-Cy7, RM4-5, Biolegend Cat#100526, 1:200
Rat monoclonal anti-mouse CD4, FITC, RM4-5, eBioscience Cat#11-0042-85, 1:200
Rat monoclonal anti-mouse CD4, Pacific Blue, YTS19.1, DRFZ Inhouse Cat#-, 1:200
Rat monoclonal anti-mouse CD4, BV421, GK1.5, Biolegend Cat#100443, 1:200
Rat monoclonal anti-mouse CD4, APC-Cy7, GK1.5, BD Cat#552051, 1:200
Rat monoclonal anti-mouse CD8a, Biotin, 53-6.7, BD Cat#553029, 1:300
Rat monoclonal anti-mouse CD8a, PE, 53-6.7, BD Cat#553033, 1:200
Rat monoclonal anti-mouse CD8a, V500, 53-6.7, BD Cat#560778, 1:200
Rat monoclonal anti-mouse CD8a, BV605, 53-6.7, Biolegend Cat#100744, 1:100
Rat monoclonal anti-mouse CD8a, BV786, 53-6.7, Biolegend Cat#100750, 1:100
Rat monoclonal anti-mouse CD8a, PerCP-Cyanine5.5, 53-6.7, eBioscience Cat#45-0081-82, 1:200
Rat monoclonal anti-mouse CD8a, eFluor450, 53-6.7, eBioscience Cat#48-0081-82, 1:200
Hamster monoclonal anti-mouse TCR ß Chain, A488, H57-597, Biolegend Cat#109215, 1:200
Hamster monoclonal anti-mouse TCR ß Chain, BV510, H57-597, BD Cat#563221, 1:200
Rat monoclonal anti-mouse IL18R, PE, P3TUNYA, eBioscience, Cat#12-5183-82, 1:200
Rat monoclonal anti-mouse TCR Va2, FITC, B20.1, BD Cat#553288, 1:100
Rat monoclonal anti-mouse CD19, Biotin, 1D3, eBioscience Cat#14-0193-85, 1:200
Rat monoclonal anti-mouse CD19, Cy5, 1D3, DRFZ Inhouse Cat#-, 1:400
Rat monoclonal anti-mouse CD19, APC-Fire750, 6D5, Biolegend Cat#115558, 1:200
Rat monoclonal anti-mouse CD19, APC, eBio1D3, eBioscience Cat#17-0193-82, 1:200
Rat monoclonal anti-mouse CD19, PerCP-Cyanine5.5, eBio1D3, eBioscience Cat#45-0193-82, 1:400
Rat monoclonal anti-mouse/human B220, Biotin, RA3-6B2, Biolegend Cat#103204, 1:100
Rat monoclonal anti-mouse/human B220, APC-Fire750, RA3-6B2, Biolegend Cat#103260, 1:200
Mouse monoclonal anti-mouse CD90.1 (Thy1.1), APC-Cy7, Ox-7 Biolegend 202520 1:200
Mouse monoclonal anti-mouse CD90.1 (Thy1.1), Cy5 Ox-7, DRFZ Inhouse - 1:400
Mouse monoclonal anti-mouse CD90.1 (Thy1.1), Pacific Blue, Ox-7, DRFZ Inhouse, Cat#-, 1:300
Rat monoclonal anti-mouse CD90.2 (Thy1.2), FITC, 53-2.1, Biolegend Cat#140304, 1:200
Rat monoclonal anti-mouse CD90.2 (Thy1.2), PE, 53-2.1, Biolegend Cat#140308, 1:200
Mouse monoclonal anti-mouse CD45.1 (Ly5.1), Pacific Blue, A20, Biolegend Cat#110722, 1:200
Mouse monoclonal anti-mouse CD45.1 (Ly5.1), FITC, A20, Biolegend Cat#110706, 1:100
Mouse monoclonal anti-mouse CD45.1 (Ly5.1), PE, A20, BD Cat#553776, 1:200
Mouse monoclonal anti-mouse CD45.2 (Ly5.2), APC, 104, BD Cat#558702, 1:100
Mouse monoclonal anti-mouse CD45.2 (Ly5.2), V500, 104, BD Cat#562129, 1:100
Mouse monoclonal anti-mouse CD45.2 (Ly5.2), APC-Fire750, 104, Biolegend Cat#109852, 1:200
Mouse monoclonal anti-mouse CD45.2 (Ly5.2), BV785, 104, Biolegend Cat#109839, 1:100
Rat monoclonal anti-mouse CD45.2 (Ly5.2), BV510, 30-F11, Biolegend Cat#103138, 1:200
Rat monoclonal anti-mouse CD44, BV421, IM7, Biolegend, Cat#103040, 1:200
Rat monoclonal anti-mouse CD44, APC-Cy7, IM7, Biolegend Cat#103028, 1:200
Rat monoclonal anti-mouse CD62L, PE-Cy7, MEL-14, eBioscience Cat#25-0621-82, 1:300
Hamster monoclonal anti-mouse KLRG1, FITC, 2F1, eBioscience Cat#11-5893-82, 1:200
Hamster monoclonal anti-mouse KLRG1, PerCP-Cyanine5.5, 2F1, Biolegend Cat#100734, 1:100
Rat monoclonal anti-mouse CD127, PE-Cy7, A7R34, eBioscience Cat#25-1271-82, 1:100
Rat monoclonal anti-mouse CD127, BV421, A7R34, Biolegend Cat#135027, 1:100
Mouse monoclonal anti-mouse NK1.1, Biotin, PK136, eBioscience Cat#13-5941-85, 1:500
Mouse monoclonal anti-mouse NK1.1, PE, PK136, BD Cat#557391, 1:200
Rat monoclonal anti-mouse Gr-1, Biotin, RB6-8C5, eBioscience Cat#13-5931-85, 1:500
Rat monoclonal anti-mouse Gr-1, FITC, RB6-8C5, DRFZ Inhouse Cat#-, 1:800
Rat monoclonal anti-mouse Gr-1, APC-Cy7, RB6-8C5, Biolegend Cat#108424, 1:200
Rat monoclonal anti-mouse NKp46, FITC, 29A1.4, Biolegend Cat#137606, 1:100

Rat monoclonal anti-mouse Siglec-F, PE, E50-2440, BD Cat#552126, 1:100
Rat monoclonal anti-mouse c-kit, APC, 2B8, eBioscience Cat#12-1171-82, 1:100
Hamster monoclonal anti-mouse FceRIa, FITC, MAR-1, eBioscience Cat#11-5898-82, 1:100
Hamster monoclonal anti-mouse FceRIa, APC-Cy7, MAR-1, Biolegend Cat#134326, 1:100
Rat monoclonal anti-mouse MHC Class II, Cy5, M5/114, DRFZ Inhouse Cat#-, 1:100
Hamster monoclonal anti-mouse CD11c, Biotin, HL3, eBioscience Cat#13-0114-85, 1:500
Hamster monoclonal anti-mouse CD11c, APC-Fire750, N418, Biolegend Cat#117352, 1:200
Rat monoclonal anti-mouse/human CD11b, Biotin, M1/70, eBioscience Cat#13-0112-85, 1:800
Rat monoclonal anti-mouse/human CD11b, PE-Cy7, M1/70, BD Cat#552850, 1:200
Rat monoclonal anti-mouse/human CD11b, BV421, M1/70, Biolegend Cat#101236, 1:200
Rat monoclonal anti-mouse/human CD11b, APC-Fire750, M1/70, Biolegend Cat#101262, 1:200
Rat monoclonal anti-mouse Ly6G, PerCP-Cyanine5.5, 1A8, Biolegend Cat#127616, 1:100
Rat monoclonal anti-mouse F4/80, PE-Cy7, BM8, Biolegend Cat#123114, 1:100
Rat monoclonal anti-mouse F4/80, APC-Fire750, BM8, Biolegend Cat#123152, 1:100
Hamster monoclonal anti-mouse CD69, BV421, H1.2F3, Biolegend Cat#104545, 1:100
Hamster monoclonal anti-mouse CD103, APC, 2E7, Biolegend Cat#121414, 1:100
Rat monoclonal anti-mouse ST2, BV421, DIH9, Biolegend Cat#145309, 1:100
Rat monoclonal anti-mouse ST2, PE, DIH9, Biolegend Cat#145303, 1:100
Rat monoclonal anti-mouse ST2, PE, RMST2-2, eBioscience Cat#12-9335-82, 1:100
Rat monoclonal anti-mouse ST2, Digoxigenin, DJ8, mbBioproducts, Conjugated in DRFZ Cat#1001101, 1:900
Sheep polyclonal anti-Digoxigenin Fab fragments, APC, -, Roche, Conjugated in DRFZ Cat#11214667001, 1:800
Sheep polyclonal anti-Digoxigenin Fab fragments, PE, -, Roche, Conjugated in DRFZ Cat#11214667001, 1:800
Mouse monoclonal anti-mouse/human T-bet, BV421, 4B10, Biolegend Cat#644816, 1:100
Mouse monoclonal anti-mouse/human T-bet, PE, 4B10, eBioscience Cat#12-5825-82, 1:100
Rat monoclonal anti-mouse/human GATA3, eFluor660, TWAJ, eBioscience Cat#50-9966-42, 1:100
Rat monoclonal anti-mouse FoxP3, eFluor450, FJK-16s, eBioscience Cat#48-5773-82, 1:100
Rat monoclonal anti-mouse Ki67, FITC, SolA15, eBioscience Cat#11-5698-82, 1:100
Mouse IgG1k Isotype Control, BV421, MOPC-21, Biolegend Cat#400158, 1:150
Mouse IgG1k Isotype Control, PE, P3.6.2.8.1, eBioscience Cat#12-4714-82, 1:200
Rat IgG2bk Isotype Control, eFluor660, 10H5, eBioscience Cat#50-4031-82, 1:400
Rat monoclonal anti-mouse IFN-g, eFluor450, XMG1.2, eBioscience Cat#48-7311-82, 1:100
Rat monoclonal anti-mouse IFN-g, APC, XMG1.2, Biolegend Cat#505810, 1:100
Rat monoclonal anti-mouse IL-2, APC, JES6-5H4, BD Cat#554429, 1:100
Rat monoclonal anti-mouse TNF, eFluor450, MP6-XT22, eBioscience Cat#48-7321-82, 1:100
Mouse monoclonal anti-mouse Granzyme A, PE, 3G8.5, Biolegend Cat#149704, 1:100
Mouse monoclonal anti-mouse/human Granzyme B, APC, QA16A02, Biolegend Cat#372204, 1:100
Rat monoclonal anti-mouse Perforin, PE, S16009B, Biolegend Cat#154406, 1:100
Rat monoclonal anti-mouse IL-4, PE, 11B11, eBioscience Cat#12-7041-81, 1:100
Rat monoclonal anti-mouse IL-13, A488, eBio13A, eBioscience Cat#53-7133-82, 1:200
Rat monoclonal anti-mouse IL-10, APC, JES5-16E3, eBioscience Cat#17-7101-82, 1:100
TotalSeq-C0301 anti-mouse Hashtag 1, Barcoded, M1/42; 30-F11, Biolegend Cat#155861, 1:200
TotalSeq-C0302 anti-mouse Hashtag 2, Barcoded, M1/42; 30-F11, Biolegend Cat#155863, 1:200
TotalSeq-C0303 anti-mouse Hashtag 3, Barcoded, M1/42; 30-F11, Biolegend Cat#155865, 1:200
a-Galactosylceramide-loaded CD1d-Tetramers, PE, -, MBL Cat#TS-MCG-1, 1:50
LCMV GP33-41 peptide-loaded MHC class I (H2-Db) tetramers, PE, -, MBL Cat#TB-M512-1, 1:50
LCMV NP396-404 peptide-loaded MHC class I (H2-Db) tetramers, APC, -, NIH Tetramer Core facility Cat#-, 1:50
Mouse monoclonal anti-human IFN-g, V450, B27, BD Cat#560372, 1:100
Mouse monoclonal anti-human IL-13, FITC, PVM13-1, ebioscience Cat#11-7139-42, 1:40
Mouse monoclonal anti-human CXCR3, FITC, G025H7, Biolegend Cat#353704, 1:50
Mouse monoclonal anti-human CD45RA, PE, HI100, DRFZ Inhouse Cat#-, 1:100
Rat monoclonal anti-human CRTH2, Biotin, BM16, Miltenyi Biotec Cat#130-113-599, 1:10
Human monoclonal anti-human CD56, APC, REA196, Miltenyi Biotec Cat#130-113-310, 1:50
Mouse monoclonal anti-human CD4, Pacific Blue, TT1, DRFZ Inhouse Cat#-, 1:200
Mouse monoclonal anti-human CD62L, PE-Cy7, DREG-56, Biolegend Cat#304822, 1:300
Mouse monoclonal anti-human CD8, FITC, GN11/134.7, DRFZ Inhouse Cat#-, 1:100
Zombie UV fixable viability kit, -, -, BioLegend Cat#423108, 1:200
Live/Dead Fixable Near-IR Dead Cell stain kit, -, -, Invitrogen Cat#L34976, 1:500
Rat monoclonal anti-LCMV nucleoprotein, -, VL-4, DRFZ Inhouse Cat#-, 1:1000
Goat polyclonal anti-Rat IgG, HRP, -, Jackson ImmunoResearch Cat#112-035-003, 1:750
Faser Kit PE, PE, -, Miltenyi Biotec Cat#130-091-764, -
Faser Kit APC, APC, -, Miltenyi Biotec Cat#130-091-762, -
Strepdavidin, PE, -, BD Cat#554061, 1:400

Antibodies used for in vitro cultures (Antibody, Clone, Provider and #Cat, Concentration):
Hamster monoclonal anti-mouse CD3e, 145-2C11, eBioscience Cat#16-0031-86, 2.5 μg/ml
Hamster monoclonal anti-mouse CD28, 37.51, eBioscience Cat#12-0281-82, 2.5 μg/ml
Rat monoclonal anti-mouse IL-4, 11B11, DRFZ Inhouse Cat#-, 10 μg/ml
Rat monoclonal anti-mouse IFN-g, XMG1.2, DRFZ Inhouse Cat#-, 10 μg/ml
Rat monoclonal anti-mouse IL-12, C18.2, DRFZ Inhouse Cat#-, 10 μg/ml
Mouse monoclonal anti-human CD3e, OKT3, Miltenyi Biotec Cat#130-093-387, 2.5 μg/ml
Mouse monoclonal anti-human CD28, 15E8, Miltenyi Biotec Cat#130-093-386, 2.5 μg/ml
Mouse monoclonal anti-human IFN-g, 45-15, Miltenyi Biotec Cat#130-095-743, 10 μg/ml
Mouse monoclonal anti-human IL-4, 7A3-3, Miltenyi Biotec Cat#130-095-753, 10 μg/ml
Mouse monoclonal anti-human IL-12, C8.6, Miltenyi Biotec Cat#130-095-755, 10 μg/ml

Validation

Commercially available antibodies have been validated by the manufacturers:

-Biolegend: https://www.biolegend.com/nl-be/quality/quality-control

Flow Cytometry Reagents
Specificity testing of 1-3 target cell types with either single- or multi-color analysis (including positive and negative cell types).
Once specificity is confirmed, each new lot must perform with similar intensity to the in-date reference lot. Brightness (MFI) is evaluated from both positive and negative populations.
Each lot product is validated by QC testing with a series of titration dilutions.

TotalSeq™ Antibodies
Bulk lots are tested by PCR and sequencing to confirm the oligonucleotide barcodes. They are also tested by flow cytometry to ensure the antibodies recognize the proper cell populations.
Bottled lots are tested by PCR and sequencing to confirm the oligonucleotide barcodes.

- eBioscience: https://www.thermofisher.com/de/de/home/life-science/antibodies/invitrogen-antibody-validation.html

Part 1—Target specificity verification
This helps ensure the antibody will bind to the correct target. Our antibodies are being tested using at least one of the following methods to ensure proper functionality in researcher's experiments:
Knockout—expression testing using CRISPR-Cas9 cell models
Knockdown—expression testing using RNAi to knockdown gene of interest
Independent antibody verification (IAV)—measurement of target expression is performed using two differentially raised antibodies recognizing the same protein target
Cell treatment—detecting downstream events following cell treatment
Relative expression—using naturally occurring variable expression to confirm specificity
Neutralization—functional blocking of protein activity by antibody binding
Peptide array—using arrays to test reactivity against known protein modifications
SNAP-ChIP™—using SNAP-ChIP to test reactivity against known protein modifications
Immunoprecipitation-Mass Spectrometry (IP-MS)—testing using immunoprecipitation followed by mass spectrometry to identify antibody targets

Part 2—Functional application validation
These tests help ensure the antibody works in a particular application(s) of interest, which may include
(but are not limited to):
Western blotting
Flow cytometry
ChIP
Immunofluorescence imaging
Immunohistochemistry

Most antibodies were developed with specific applications in mind. Testing that an antibody generates acceptable results in a specific application is the second part of confirming antibody performance.

- Miltenyi Biotec: https://www.miltenyibiotec.com/DE-en/products/macs-antibodies/antibody-validation.html
During development of an antibody, a suitable test to verify specificity of the clone is performed. Several approaches are possible (Counterstaining, Knockout of protein, Epitope competition assay, siRNA knockdown of protein, Stimulation of cells, Overexpression of target protein, Binding to purified antigen)

-BD Biosciences: https://www.biocompare.com/Antibody-Manufacturing/355107-Antibody-Manufacturing-Perspectives-BD-Bioscience/
We conduct quality control (QC) testing in primary model systems to ensure biological accuracy in an ISO 9001 certified facility. BD conducts rigorous QC testing of each antibody lot tested side-by-side with a previously produced lot as reference. Our product development process includes testing on a combination of primary cells, cell lines and/or transfectant cell models with relevant controls using multiple immunoassays to ensure biological accuracy. We also perform multiplexing with additional antibodies to interrogate antibody staining in multiple cell populations.

Further, several antibodies have been validated in-house by using knockout mice, unstimulated cells, or isotype antibodies as controls. Antibodies used for determination of viral titers in stock solutions were verified using virus-free samples as controls.

Eukaryotic cell lines

Policy information about cell lines

Cell line source(s)

HEK293T, MC57G, and L929 cells were obtained at ATCC. NIH3T3 fibroblasts were kindly provided by the Max Plank Institute for Infection Biology (Berlin) and originally obtained at ATCC. BHK-21 cells were obtained at ECACC.

Authentication

None of the cell lines used were additionally authenticated.

Mycoplasma contamination

Cell lines were not tested for mycoplasma contatmination.

Commonly misidentified lines (See ICLAC register)	HEK293T, MC57G, L929, NIH3T3, and BHK-21 cells are not commonly misidentified cell lines.

Animals and other organisms

Policy information about studies involving animals; ARRIVE guidelines recommended for reporting animal research

Laboratory animals	C57BL/6J mice (wildtype), LCMV-TCRtg P14 (Pircher et al. 1989) and Smarta mice (Oxenius et al. 1998) expressing the congenic markers CD45.1 (Ly5.1) or CD90.1 (Thy1.1), Il1rl1-/- (Townsend et al. 2000), Il1rl1-ExAB-/-, Il1rl1-ExC-/-, Stat4-/- (Kaplan et al. 1996), Tbx21-/- (Szabo et al. 2002), Smarta x Il1rl1-ExAB-/-, Smarta x Stat4-/-, Smarta x Tbx21-/-, P14 x Il1rl1-/-, and P14 x Il1rl1-ExAB-/- and TCRbd-/-(Mombaerts et al. 1992) mice were bred under specific-pathogen free conditions in approved animal-care facilities at the Research Institute for Experimental Medicine (FEM) of the Charité – Universitätsmedizin Berlin (Berlin, Germany) or at the Laboratory Animal Facility of the ETH Zürich (ETH Phenomics Center, Zürich, Switzerland). Mice were housed in individually ventilated cages with a 12 h light/dark cycle at an ambient temperature of 21°C and 45% to 65% relative humidity. Mice had ad libitum access to drinking water and chow. Both, male and female mice between 8 and 26 weeks of age were used for experiments. For LCMV infections, experimental groups were age- and sex-matched. Mice used for scRNA-Seq analyses were cohoused for 4 weeks prior to LCMV infection. Il1rl1-ExAB-/- and Il1rl1 ExC-/- mice were generated in the Transgenics Core Facility of the Max Delbrück Centrum Berlin.
Wild animals	The study did not use wild animals
Field-collected samples	The study did not include field-collected samples
Ethics oversight	Animal experiments were performed in accordance with institutional guidelines and the German and Swiss law for animal protection and were approved by the respective governmental authorities (Landesamt für Gesundheit und Soziales Berlin and the Cantonal Veterinary Office of the Canton of Basel; G0111/17, G0206/17, G0245/19).

Note that full information on the approval of the study protocol must also be provided in the manuscript.

Human research participants

Policy information about studies involving human research participants

Population characteristics	Due to German Data Privacy laws, no data such as gender or age are legally available for healthy blood donors from the German Red Cross. No influence of gender or age on the readout was to be expected.
Recruitment	Human peripheral blood of random healthy donors was obtained from the German Red Cross (DRK Berlin, Germany).
Ethics oversight	The Charité ethics commitee approved the study (EA1/149/12).

Note that full information on the approval of the study protocol must also be provided in the manuscript.

Flow Cytometry

Plots

Confirm that:

☒ The axis labels state the marker and fluorochrome used (e.g. CD4-FITC).

☒ The axis scales are clearly visible. Include numbers along axes only for bottom left plot of group (a 'group' is an analysis of identical markers).

☒ All plots are contour plots with outliers or pseudocolor plots.

☒ A numerical value for number of cells or percentage (with statistics) is provided.

Methodology

Sample preparation	To isolate lymphocytes, spleens were mechanically disrupted and filtered through 70 µm strainers. Erythrocytes were lysed by 3-5 min of incubation in erythrocyte lysis buffer (10 mM KHCO3, 155 mM NH4Cl, 0.1 mM EDTA, pH 7.5). Livers were collected in PBS/BSA, meshed and centrifugated at 30 g for 2 min to remove debris. Supernatants were subjected to Histopaque density centrifugation (1.083 g/ml, Sigma-Aldrich) and lymphocytes were collected at the gradient interphase. To stain ILC2s, lungs were cut into small pieces and digested with Collagenase D (0.1 U/ml) in RPMI1640 (supplemented with 10% FCS and 15mM HEPES) for 1 h at 37°C. Afterwards, lymphocytes were isolated by Histopaque density centrifugation (1.083 g/ml, Sigma-Aldrich). To isolate peritoneal cavity cells, 5 ml of cold PBS was injected into the peritoneal cavity of euthanized mice. After a brief massage of the peritoneum, cell-containing liquid was collected and subjected to Histopaque density centrifugation (1.083 g/ml, Sigma-Aldrich). For analysis of tissue-resident memory T cells, lungs, kidneys, and salivary glands were cut into pieces and digested in RMPI1640 + GlutaMax I (Thermo Scientific) medium containing FCS (5% v/v, Thermo Scientific), MgCl2 (2 µM, Carl Roth), CaCl2 (2 µM, Carl Roth), and collagenase type I (100 U/ml, Gibco) at 37°C for 45 min. Subsequently, tissue was further disrupted using a GentleMACS Dissociator (setting m_Spleen_01.01). Cells were filtered through 70 µm strainers, subjected to erythrocyte lysis, and analyzed.
Instrument	FACS Canto, LSR Fortessa (BD) and MACSQuant (Miltenyi Biotec) were used for data collection Aria I and Aria II (BD) devices were used for cell sorting.

Software	BD Diva software (v8.1), FlowJo (v10.7.1)
Cell population abundance	Frequencies of analyzed cell populations are indicated in representative plots. Purity of sorts are shown in representative plots in the Supplementary Information.
Gating strategy	Lymphocytes were defined using FSC-A and SSC-A and singlets were further selected using FSC-W vs. FSC-H and SSC-W vs. SSC-H or FSC-A vs. FSC-H. For cell sorting, dead cells were labelled with propidium iodide. For analysis of surface markers and intracellular stainings, dead cells were labelled with Zombie Aqua or Zombie NIR fixable live/dead staining reagents (Biolegend). Further gatings were defined based on bimodal expression of surface markers. Gating strategies are indicated in Figure Legends and representative plots showing gating strategies are provided in the Supplementary Information.

☒ Tick this box to confirm that a figure exemplifying the gating strategy is provided in the Supplementary Information.
