# Supplementary figures and images for: A type 1 immunity-restricted promoter of the IL−33 receptor gene directs antiviral T-cell responses
Source: Nat Immunol. 2024 Jan 3;25(2):256–67. doi: 10.1038/s41590-023-01697-6 (PMC10834369; doi:10.1038/s41590-023-01697-6)

PCR Gel pictures Figure 1f

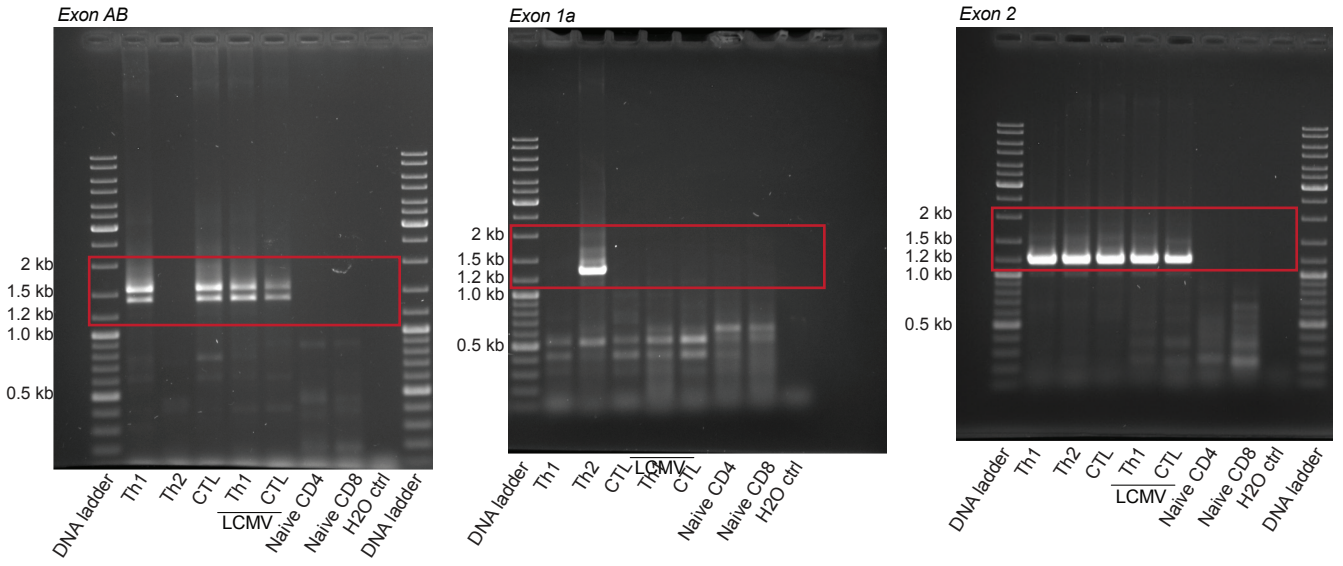

Supplement: Supplementary file 5 — Unprocessed gels. [file 41590_2023_1697_MOESM5_ESM.pdf]

Uncropped FACS plots Figure 3b

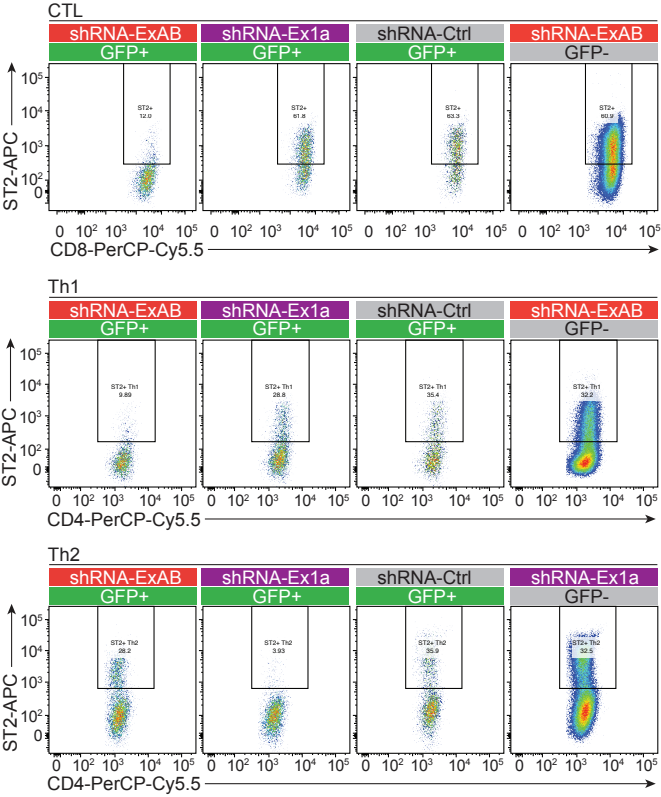

Supplement: Supplementary file 8 — Unprocessed FACS plots. [file 41590_2023_1697_MOESM8_ESM.pdf]

PCR Gel pictures Extended Data Figure 3c

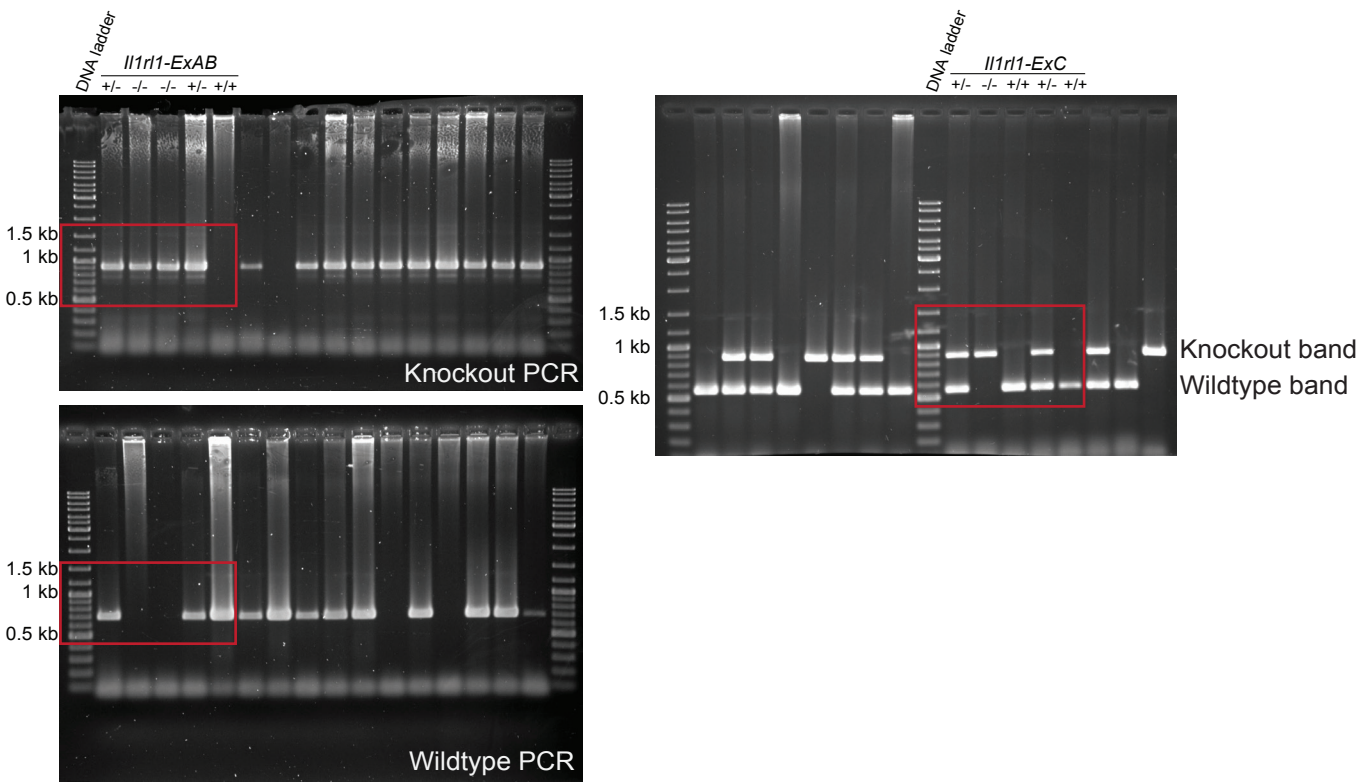

Supplement: Supplementary file 15 — Unprocessed gels. [file 41590_2023_1697_MOESM15_ESM.pdf]

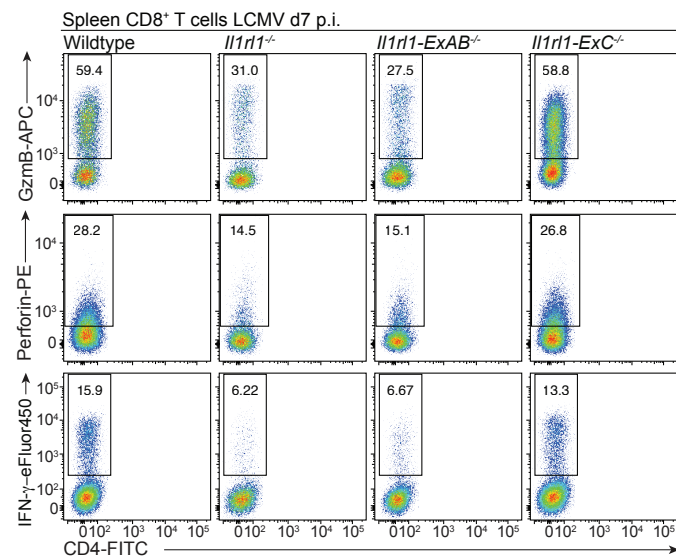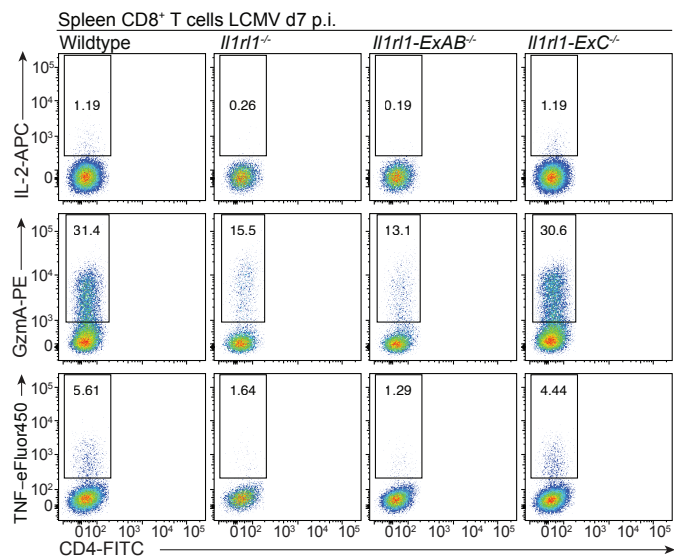

Supplement: Supplementary file 19 — Unprocessed FACS plots. [file 41590_2023_1697_MOESM19_ESM.pdf]
